# Development and Application of the Owner-Bird Relationship Scale (OBRS) to Assess the Relation of Humans to Their Pet Birds

**DOI:** 10.3389/fvets.2020.575221

**Published:** 2020-12-10

**Authors:** Anne-Kathrin Burmeister, Katrin Drasch, Monika Rinder, Sebastian Prechsl, Andrea Peschel, Rüdiger Korbel, Nicole J. Saam

**Affiliations:** ^1^Center for Clinical Veterinary Medicine, Clinic for Birds, Small Mammals, Reptiles and Ornamental Fish, Ludwig-Maximilians-Universität München, München, Germany; ^2^Institute of Sociology, Friedrich-Alexander-Universität Erlangen-Nürnberg, Erlangen, Germany

**Keywords:** human-animal relationship, pet birds, companion bird, scale, anthropomorphism, social support (MeSH term)

## Abstract

Only a few birds besides domestic pigeons and poultry can be described as domesticated. Therefore, keeping a pet bird can be challenging, and the human-avian relationship will have a major influence on the quality of this cohabitation. Studies that focus on characterizing the owner-bird relationship generally use adapted cat/dog scales which may not identify its specific features. Following a sociological approach, a concept of human-animal relationship was developed leading to three types of human-animal relationship (impersonal, personal, and close personal). This concept was used to develop a 21-item owner-bird-relationship scale (OBRS). This scale was applied to measure the relationship between pet bird owners (or keepers) (*n* = 1,444) and their birds in an online survey performed in Germany. Factor analysis revealed that the relationship between owner and bird consisted of four dimensions: the tendency of the owner to anthropomorphize the bird; the social support the bird provides for the owner; the empathy, attentiveness, and respect of the owner toward the bird; and the relationship of the bird toward the owner. More than one quarter of the German bird owners of this sample showed an impersonal, half a personal, and less than a quarter a close personal relationship to their bird. The relationship varied with the socio-demographic characteristics of the owners, such as gender, marital status, and education. This scale supports more comprehensive quantitative research into the human-bird relationship in the broad field of human-animal studies including the psychology and sociology of animals as well as animal welfare and veterinary medicine.

## Introduction

Humans and birds have lived together from time immemorial. In the beginning, birds were most likely kept for food. According to Xiang et al. (1), the domestication of chickens probably started about 10,000 years ago. However, exactly when humans started to keep birds as companion animals cannot be determined, although caring for parrots can be traced back at least 2,500 years (2, 3). Despite this long history, one problem of bird-keeping in modern times is the difficulty of domesticating them. Besides domestic pigeons and poultry, only a few pet birds can be described as domesticated (4), for example budgerigars and cockatiels (5), canaries (6), and Bengalese finches (7). Important behavior, such as flight, are often reflexive (8). Therefore, living with a bird can be challenging, and the owner (used here synonymous to the keeper) has to learn how to give the bird the best possible life in captivity (9). The quality of this cohabitation is influenced by the human-avian relationship. Yet to date, only a few studies have focused on characterization of this relationship.

Previous studies investigating the human-avian relationship focused on qualitative aspects of this relationship or attempted to find differences between bird owners and owners of other companion animals. These studies were based on general pet attachment scales, such as the “Pet attachment survey” (PAS) (10) and the “Lexington Attachment to Pets Scale” (LAPS) (11). Beck and Katcher (12) using PAS included in a questionnaire survey with 42 bird owners as well as interviews and observations of 18 persons, found that interaction with a bird was similar to that with dogs or cats and involved “talk, touch, care giving, and the assumption of real communication” (12). Using LAPS, Bennett and O'Hara (13) revealed that parrot owners were as strongly attached to their birds as owners of other pets. Avian companions met the psychological needs of humans and provided social support or fulfilled esteem and cognitive needs (14). A survey by Kidd and Kidd (15) based on interviews and focusing on personality factors of bird owners revealed that interactions were close and loving when the owners were patient, warm, caring, and curious. Friendship, companionship, and verbal interaction were the main benefits of bird-keeping, while messiness and the noise of the birds were the main problems that keepers experienced.

Further studies have considered the personal characteristics of the owners, the benefits of keeping birds and the influence of the owners on the birds' welfare and special needs. Central results of these earlier studies were that the participants of quantitative studies described themselves as low self-monitoring individuals (16), and that they were socially outgoing and expressive (17). Two studies focusing on parrots kept as companion birds combined qualitative and quantitative research and described the human-parrot bond by evaluating owner essays. When asked about the most rewarding aspects of avian companionship, most parrot keepers mentioned “love,” “birds as family or ‘Fids’ (Feathered Kids),” “talking ability,” and “companionship” (9). Parrots provided social support leading to mental and physical benefits of the owner. The human-companion parrot bond was described to have sometimes also negative effects on the bird when, for example, situational anthropomorphism lead to a misinterpretation of parrot behavior or when anthropocentrism reduced the bird to an object (18).

While all these studies provided important qualitative insights into the multiple facets of the human-bird relationship, none of them involved a scale to measure the relation of humans to birds and to identify its specific features. An uncritical application of scales developed for dogs or cats might, however, result in an incorrect assessment of the quality or intensity of the human-bird relation and lead to wrong conclusions. Problems even arose when scales constructed for dogs were used for measuring the human-cat relation. While the Comfort from Companion Animal Scale (CCAS) was developed and pretested, dog owners seemed to have a significantly higher degree of attachment than cat owners when the scale included 2 items pertaining to dogs. Use of 11 items referring to emotional features of the human-pet relationship, however, revealed no differences in attachment levels in the 2 owner groups (19). Behaviors mainly pertaining to dogs referred to various physical and interactive activities like taking walks, traveling together, grooming, or training the animal. Emotional aspects of the relationship such as the love, trust, loyalty, and joyful mutual activity were, in contrast, considered to be useful to characterize relationships of humans with a broad range of (or even all) pets (19). Qualitative observations of owners of birds (primarily parakeets and parrots) showed both similarities and differences of interactions compared with interactions with dogs and cats. Similarities included human facial expression and voice inflection during interactions as well as a particular loyalty of the pet to one or two specific people. In addition, a kind of jealousy the pet showed when attention of the owner was diverted away was also regarded as similar in birds, dogs and cats. Bird-owner interactions were described to include the peculiarities that bird owners needed more efforts to elicit a positive response and more time to train the bird to accept human contact. Further observations of differences included, that it was more difficult for owners to physically interact with birds and that “bird talking” was perceived very satisfying (12). It has to be noted that the just mentioned particularities of the bird- human interaction especially pertain to parrots and parakeets but not to all birds equally. It is thus important to follow a multifaceted approach to understand the nature of different types of human–animal relationships (20).

A bird-specific scale is thus a prerequisite for more comprehensive quantitative research into the human-bird relationship and is especially needed to detect peculiarities of this relationship with regard to bird species groups. “A dog is not a cat is not a bird” (18, 19). This insight from Zasloff's study on measuring attachment to companion animals (19), coupled with the assumption of Hergovich et al. (21) that attachment to exotic pets might differ both in quantity and in quality from attachment to traditional pets and coupled with the argument by Bergler et al. (22) that the human-animal relationship always depends on the species as well as on the breed and individual type, motivated us to develop a scale to measure the owner-bird relationship and to validate the scale in an online survey.

### A Concept of Human-Animal Relationship

In accordance with the standards of quantitative methods of empirical social research, which require that the different dimensions of a concept are specified with reference to theory (23), a concept of human-animal relationship was developed to be taken as a basis to operationalize the owner-bird relationship.

Following a sociological approach (24–26) based on the sociology of close relationships (27) and symbolic interactionism (28), a concept of human-animal relationship relying on five dimensions is proposed: (1) series of interactions, (2) personal identity, (3) reciprocity, (4) emotional bond, and (5) empathy. Three types of human-animal relationship can be distinguished. An *impersonal human-animal relationship* is characterized by a series of interactions between a human and an animal that do not recognize each other reciprocally, although unilateral recognition is possible. The interactions are influenced by the human's social role and the animal's needs (e.g., the treatment of a parrot by a veterinarian). A *personal human-animal relationship* is distinguished by a series of interactions between a human and an animal that do recognize each other reciprocally: every interaction is influenced by the history of previous interactions and by the expectations for future interactions. Both parties have personal knowledge about each other that can, however, be different in degree (e.g., a bird owner feeding her finches). Finally, a *close personal human-animal relationship* is understood as a personal human-animal relationship additionally characterized by a high degree of reciprocity. The human and the animal adjust their behavior to each other and show empathy for one another. Specific norms and habits exist that characterize the uniqueness of this relationship. The animal and the human develop an emotional bond (e.g., a child playing with and cuddling her budgie) (Table 1). A special type of close personal human-animal relationship was designated as the human-animal friendship (25).

**Table 1:** Concept of three types of human-animal relationships.

**Dimension**	**Impersonal human-animal relationship**	**Personal human-animal relationship**	**Close personal human-animal relationship**
Series of interactions	x	x	x
Personal identity		x	x
Reciprocity			x
Emotional bond			x
Empathy			x

*x, dimension is a component of the type of human-animal relationship*.

It is, at present, unknown whether all these dimensions of the concept of human-animal relationship (Table 1) are also part of the relationships of humans to their pet birds. Therefore, the aim of this investigation was to develop an owner-bird relationship scale based on this concept. Application of this new scale should reveal qualities and quantities of dimensions occurring in the relationships of humans to their pet birds and should allow their standardized measurement.

## Materials and Methods

A multi-step process was used to develop the owner-bird relationship scale (OBRS). Several scales developed for dogs and cats (see below) were reviewed and adapted. Next, a range of further questions and items for a questionnaire were developed to cover missing dimensions. The final questionnaire was applied as an online survey and obtained data have been analyzed.

### Questionnaire Design

In order to measure the owner-bird-relationship a questionnaire was designed during a four-step procedure. The questions and items for the questionnaire were developed in a focus group by an interdisciplinary team of four veterinarians and one sociologist, all of whom were familiar with human-bird relationships in a professional or private capacity. The data were collected in Germany, and therefore all items and questions were prepared in German.

In a first step, 52 items were deduced from several scales developed for dogs and cats (11, 29–40) and modified for pet birds. Some items referring to physical activities such as “I take my pet along when I go jogging or walking” (33) have been omitted, others have been adjusted for pet birds. Sometimes, only the word “pet” was replaced by “bird,” while in other cases, a more distinct modification of the original item was performed. For example the item “Birds are sentient or aware beings with thoughts and feelings of their own” (18) was modified to “My bird is a sensitive being with its own needs. (Mein Vogel ist ein empfindsames Lebewesen mit seinen eigenen Bedürfnissen).” A five-point Likert scale was employed measuring (dis)agreement ranging from “strongly disagree” (1) to “strongly agree” (5). The scale was included in a short questionnaire together with questions about each bird's species and the owner's socio-demographic characteristics.

In a second step, this preliminary scale was tested as a paper-and-pencil self-administered questionnaire (pre-study). The questionnaire was distributed to the clients of two veterinary clinics, to veterinary students, and to visitors of bird exhibitions. Data were collected from July to November 2014. 294 bird owners (166 females, 119 males, 9 unknown) participated in the pre-study. A principal component analysis (PCA) (41) was performed to receive information on the common structure of the 52 items and to identify redundant items that could be deleted in the main study. Based on this analysis, a total of 23 items were retained.

In a third step, a critical review of the meaning of the identified factors revealed that not all five human-animal relationship dimensions (series of interactions, personal identity, empathy, reciprocity, emotional bond, Table 1) were captured. Therefore, a more comprehensive scale covering also the missing dimensions was developed. The 23 items obtained from the pre-study and mentioned above in the second step were used, adjustments were made to the wording of some of those items. Moreover, 16 items were newly developed for the missing dimensions. Items to measure social support and items to measure reciprocity were now included. We also decided to ask for the proximity or distance-seeking behavior of the bird toward the owner. These items were deduced from the stationary person test which is used to measure approach and avoidance behavior in poultry as a feature of the chicken-human relationship (42). In this way, shy, distance-appreciating birds and birds that seek close contact with the owner were distinguished. The five-point Likert scale was retained. The final questionnaire thus included a scale containing 39 items covering the five human-animal relationship dimensions mentioned above as well as items measuring social support and the behavior of the birds toward the owner. The affiliation of the final 21 OBRS items to these categories, as well as the original scales the items were deduced from, are listed in Table 2. Sections on the bird owner's biography in pet-keeping, information on the species, the housing of the bird, the behavior, and the socialization of the bird as well as the perceived importance of a specialized avian veterinarian were also included in the questionnaire. The complete questionnaire is available from the authors on request.

**Table 2:** Origins of the final 21 items of the owner-bird-relationship scale (OBRS) (translated in English, the original German formulation is given in parentheses).

**Item No**.	**Questionnaire item**	**Scale of origin**	**Item references**
1	I enjoy playing with my bird (Ich spiele gerne mit meinem Vogel).	LAPS	I play with my pet quite often (11).
2	I think my bird understands me (Ich bin der Meinung, dass mein Vogel mich versteht).	LAPS	My pet understands me (11)
3	My bird knows when I'm feeling bad (Mein Vogel weiß, wann es mir schlecht geht).	LAPS	My pet knows when I'm feeling bad (11).
4	I consider my bird to be a friend (Ich betrachte meinen Vogel als einen Freund).	LAPS	I believe my pet is my best friend (11).
5	My bird is an equal member of my family (Mein Vogel ist ein gleichberechtigter Teil meiner Familie).	PRS	My pet is an equal in this family (33).
6	Sometimes I wonder what my bird is thinking (Manchmal frage ich mich, was mein Vogel wohl gerade denkt).	OBRS	This study
7	I can talk to my bird about anything (Mit meinem Vogel kann ich über alles reden).	PRS	I talk to my pet about things that bother me (33)
8	My bird is like a child to me (Mein Vogel ist wie ein Kind für mich).	No name given to scale	Deduced from an open essay question. Parrots were described as feathered kids (9).
9	My bird provides structure for my life (Mein Vogel gibt meinem Leben eine Struktur).	CCAS	My pet is a source of constancy in my life (19).
10	Having a bird gives me something to care for (Einen Vogel zu besitzen gibt mir etwas, um das ich mich kümmern kann).	CCAS	Having a pet gives me something to care for (19).
11	My bird makes me feel needed (Durch meinen Vogel fühle ich mich gebraucht).	CCAS	My pet makes me feel needed (19).
12	Spending time with my bird makes me forget my problems for a while (Mich mit meinem Vogel zu beschäftigen, lenkt mich von meinen Problemen ab).	No name given to scale	Watching the birds at the feeder makes me forget my problems for a while (43).
13	I feel relaxed/more content because of my bird (Durch meinen Vogel bin ich ausgeglichener und zufriedener).	OBRS	This study
14	I feel distressed when my bird is ill and I see it suffering (Es belastet mich, wenn mein Vogel krank ist und ich ihn leiden sehe).	OBRS	This study
15	When my bird is ill, it is my duty to care for it (Wenn mein Vogel krank ist, ist es meine Pflicht, mich um ihn zu kümmern).	No name given to scale	I feel responsible for the well-being of the birds (43).
16	I pay attention to my bird's body language (Ich achte auf die Körpersprache meines Vogels).	No name given to scale	I understand my bird's natural body language and vocalizations (18).
17	My bird has its own unique personality (Mein Vogel hat seine ganz eigene Persönlichkeit).	OBRS	This study
18	My bird is a sensitive being with its own needs (Mein Vogel ist ein empfindsames Lebewesen mit seinen eigenen Bedürfnissen).	No name given to scale	Birds are sentient beings with thoughts and feelings (18).
19	My bird actively tries to be close to me (Mein Vogel sucht von sich aus meine Nähe).	OPR	My pet enjoys my company (44).
20	My bird always keeps a little distance from me (Mein Vogel hält immer ein bisschen Abstand von mir).	OBRS	This study
21	My bird ignores me (Eigentlich ignoriert mich mein Vogel).	OBRS	This study

*Scales of origin: CCAS, Comfort from Companion Animal Scale; LAPS, Lexington Attachment to Pet Scale; OBRS, Owner-Bird Relationship Scale; OPR, Owner-Pet Relationship Scale; PAS, Pet Attachment Survey, PRS, Pet Relationship Scale*.

In a fourth step, this questionnaire was pre-tested with a sample of *n* = 18 participants to identify difficulties, ambiguous items, or other problems in the questionnaire. Some questions were reworded according to remarks made by participants of the pre-test, as, for example, the question on the total number of birds owned by the participants.

It is pointed out here that we gave instructions to the participants pertaining to the bird: because a bird keeper may own several birds, but is required by the questionnaire to answer for only one of them, the choice of bird may be the result of several sources of bias. For example, the bird owner might decide to answer the questions for a favorite bird. To avoid this, the bird owner was asked either to choose the older of two birds if he/she owns two or to choose the median-aged bird. Since selection of a median-aged birds might be difficult, the following instruction were added: The respondents were asked to imagine their birds sitting sorted by their age on a limb of a tree and then pick the middle one or the one sitting approximately in the middle. This instruction served as proxy for a random rule to choose the bird. We did not apply the random rule suggested by Delgado and Reevy (45), who proposed choosing the animal whose name begins with a letter closest to the letter “A.” This rule is not generally suitable for bird selection because many birds are not given names, especially when kept in large groups.

### Data Collection

The study was conducted as an online survey using *EFS Survey*. Data were collected from August to October 2015. The survey link to the questionnaire of the main study was distributed to bird owners throughout Germany who were contacted through several sources via the snowball sampling technique. This nonprobability sampling technique was used to reach as many bird owners as possible because of the unavailability of any appropriate data base, such as a list of registered bird owners in Germany. Participants were reached via the internet (social networks, internet forums, web sites, and email discussion groups—all of them about birds) and conventional methods (veterinary clinics, zoo shops, bird journals, and in-person groups to acquaint bird owners with the project) with a request for cross posting and an internet link.

Data collection, storage, and processing was done in accordance with the German data protection laws then in force. After clicking on the link, prospective participants were given information about the purpose of the study and issues of data protection on the first page of the online questionnaire. Informed consent had to be given actively before the survey could be started. Participation was voluntary. Data collection was anonymous and no personal nor other sensible data were collected. The survey could be terminated at any point. Therefore, no approval by an ethics committee was required as per the local legislation. The regulations and requirements of the data collection platform Unipark and the university concerning how the data can be distributed and collected were met.

### Participants

The participants comprised 1,444 bird owners (1,092 females, 351 males, 1 unknown) ranging in age from 16 to 99 years (mean: 40 years). Descriptive data regarding their socio-demographic characteristics, such as gender, education, net household income, marital status, and residential area, can be found in the final column of Table 3. Note that “region” refers to the current state of residence of the respondent and was dichotomized to East and West Germany. Although the German reunification took place in 1989, there are still numerous research results that show substantial differences between East (former socialist GDR) and West (FRG) that are often difficult to explain. Because we were not sure whether differences might exist in our case, we included this variable as a control. We decided to include Berlin in West Germany, potentially creating a small empirical error. The number of birds per participant varied: 4% of the participants owned only one bird, 21% owned two of them, and 75% had three or more pet birds. It has to be noted that according to animal protection regulations, birds of most species included in this investigation should not be kept as single birds (the exception being raptors who are a rather small group (*N* = 36) in our sample). We aggregated bird groups according to their empirical distribution and distinguished between parrots and parakeets, finches (birds of the families Fringillidae and Estrildidae as well as other small finch-like birds like weaver finches), ornamental fowl, and others (Table 3). We did not impute missing values due to item nonresponse on these demographics. Therefore, the number of cases available for each variable is different.

**Table 3:** Average factor scores by respondent demographics and bird group.

	**Factor 1 “Bird as human”**	**Factor 2 “Social support”**	**Factor 3 “Empathy, attentiveness, and respect”**	**Factor 4 “relationship of the bird toward the owner”**	***N* (%)**
**Gender**	^***^		^***^		
Female	0.10^a^ (0.97)	−0.00 (0.98)	0.11^a^ (0.92)	0.02 (1.00)	1,010 (76%)
Male	−0.35^b^ (1.03)	−0.01 (1.07)	−0.35^b^ (1.18)	−0.07 (1.00)	314 (24%)
**Age**	^**^	^***^	^**^		
≤25	0.21^a^ (0.86)	0.26^a^ (0.86)	0.04^a^ (0.94)	−0.08 (0.96)	203 (15%)
26-45	−0.01^a,b^ (0.99)	0.04^a,b^ (0.96)	0.01^a^ (0.97)	−0.01 (1.03)	633 (48%)
46-65	−0.09^a,b^ (1.07)	−0.16^b^ (1.06)	0.04^a^ (0.93)	0.04 (0.99)	444 (34%)
≥66	−0.24^b^ (0.89)	−0.11^b^ (1.29)	−0.61^b^ (1.52)	0.22 (0.83)	33 (3%)
**Education**	^***^	^***^	^*^		
Tertiary education	−0.26^a^ (0.88)	−0.25^a^ (0.96)	0.12^a^ (0.90)	−0.03 (1.06)	340 (27%)
Vocational training	0.02^b^ (1.04)	0.07^b^ (1.02)	−0.01^a,b^ (0.98)	−0.00 (0.99)	758 (60%)
Without vocational training	0.23^c^ (0.98)	0.18^b^ (0.90)	−0.10^b^ (1.23)	0.04 (0.93)	163 (13%)
**Income**	^***^	^***^		^**^	
below 1,000 €	0.25^a^ (0.83)	0.22^a^ (0.94)	0.07 (1.06)	−0.37^a^ (1.16)	103 (10%)
1,000 up to below 2,000 €	0.19^a, b^ (0.96)	0.24^a^ (0.85)	0.03 (0.80)	0.02^a, b^ (0.98)	281 (27%)
2,000 up to below 3,000 €	0.02^a, b, c^ (1.03)	0.04^a, b^ (0.97)	−0.05 (0.94)	0.11^b^ (1.00)	259 (25%)
3,000 up to below 4,000 €	−0.26^c^ (0.94)	−0.02^a, b, c^ (0.99)	0.02 (0.87)	−0.10^a, b^ (0.97)	187 (18%)
4,000 up to below 5,000 €	−0.16^b, c^ (1.00)	−0.23^b, c^ (1.12)	0.01 (1.17)	−0.00^a, b^ (0.98)	95 (9%)
5,000 € and more	−0.25^c^ (0.97)	−0.36^c^ (1.05)	−0.14 (1.36)	0.07^b^ (1.01)	126 (12%)
**Marital status**	^***^	^***^		^**^	
Married	−0.14^a^ (1.06)	−0.10^a^ (1.05)	−0.04 (1.03)	0.08^a^ (0.98)	677 (54%)
Unmarried	0.13^b^ (0.91)	0.15^b^ (0.90)	0.05 (0.91)	−0.08^b^ (1.01)	567 (46%)
**Region**			^*^	^*^	
West Germany	−0.01 (1.00)	−0.02 (0.99)	0.03 (0.99)	0.02^a^ (1.00)	1,153 (87%)
East Germany	−0.01 (1.01)	0.06 (1.07)	−0.16^b^ (1.06)	−0.17^b^ (1.01)	172 (13%)
**Residential area**	^***^		^*^		
Urban	0.14^a^ (0.97)	−0.02 (1.00)	0.05^a^ (1.01)	−0.05 (1.02)	778 (59%)
Rural	−0.23^b^ (1.01)	0.02 (1.01)	−0.07^b^ (0.99)	0.057 (0.98)	531 (41%)
**Bird group**	^***^	^**^	^***^	^***^	
Parrots and parakeets	0.36^a^ (0.86)	−0.07^a^ (1.01)	0.16^a^ (0.85)	−0.00^b^ (1.02)	810 (61%)
Finches	−0.44^b, c^ (1.01)	0.12^a, b^ (0.96)	−0.32^b^ (1.15)	−0.65^a^ (0.94)	104 (8%)
Ornamental fowl	−0.69^b^ (0.88)	0.03^a, b^ (0.96)	−0.27^b^ (1.22)	0.15^b^ (0.92)	324 (24%)
Other	−0.32^c^ (0.97)	0.34^b^ (1.00)	−0.06^a, b^ (0.92)	0.19^b^ (0.91)	87 (7%)
Gender^*^bird group	^**^		^***^		
**Men (314)**					
**Bird group**					
Parrots and parakeets	0.04^a^ (0.95)	−0.18^a^ (1.05)	0.03^a^ (0.79)	−0.06^b^ (1.06)	148 (47%)
Finches	−0.89^b^ (0.94)	0.18^a, b^ (0.91)	−0.84^b^ (1.32)	−0.69^a^ (0.88)	46 (15%)
Ornamental fowl	−0.76^b^ (1.00)	−0.10^a^ (1.13)	−0.86^b^ (1.54)	0.06^b^ (0.92)	70 (22%)
Other	−0.41^a, b^ (0.93)	0.43^b^ (1.07)	−0.28^a^ (1.00)	0.30^b^ (0.76)	50 (16%)
**Women(1,010)**					
**Bird group**					
Parrots and parakeets	0.43^a^ (0.83)	−0.05 (1.00)	0.19 (0.86)	0.01^a^ (1.01)	661 (65%)
Finches	−0.08^b^ (0.92)	0.08 (1.00)	0.12 (0.78)	−0.62^b^ (0.99)	58 (6%)
Ornamental fowl	−0.68^c^ (0.84)	0.06 (0.90)	−0.11 (1.07)	0.18^a^ (0.93)	254 (25%)
Other	−0.20^b^ (1.02)	0.20 (1.00)	0.23 (0.71)	0.04^a^ (1.07)	37 (4%)

*Means, standard deviations (in parentheses), z-standardized*.

* *p < 0.05*,

** *p < 0.01*,

*** *p < 0.001. Mean values with different superscripts differ significantly on the basis of Scheffé ANOVA post-hoc tests; or, in other words, homogeneous groups share the same superscript*.

### Data Analysis

Descriptive statistics such as means and standard deviations of the variables of interest were calculated using Stata. Principal component analysis (PCA) (41) without orthogonal rotation was used to detect an item structure. Then, PCA with orthogonal rotation was used to explore and identify the dimensions of the OBRS. For both procedures Stata and SPSS was used. Simple structure was achieved with varimax rotation that aims at achieving high loadings on one component, with minimal loadings on the others. Reporting the varimax is appropriate when using exploratory PCA if the goal is to achieve simple structure and ease of interpreting the rotated matrix (46). Factors loading above 1 were extracted following the Eigenvalue criterion (which suggests interpreting all factors loading above 1). Cronbach's alpha was used to calculate the internal consistency of the items of each of the factors as well as of the complete scale. In addition, the percentage of explained variance was calculated to determine the quality of the factor. All statistical tests were evaluated against *p* < 0.05 or otherwise tagged. The factor analysis included *N* = 1,325 individuals with non-missing information with respect to the OBRS items. One hundred and nineteen individuals had to be excluded because they had incompletely answered the OBRS items.

As the developed factors were approximately normally distributed, analysis of variance (ANOVA) techniques were applied to establish differences between the means of the extracted factors and other central variables. In order to examine differences between the means, multiple comparisons were used. Although not all variances were homogeneous, Scheffé's method was applied to compare multiple groups since this method has been described as robust against moderate violations of the homogeneity of variances assumption and is able to handle unbalanced data with different group sizes (47, 48).

Robustness checks were also conducted using only the largest group of bird species (budgies). These analyses reveal the same factors. Because of the limited number of cases for the other bird species, factor analyses for those subgroups were not informative.

## Results

### Factors Characterizing the Human-Bird Relationship

The item pool initially consisted of 39 items. However, based on the results of the principal component analysis (PCA) (41), five items as confounders were removed because they correlated positively as well as negatively with other items. Another seven items were removed because they did not discriminate sufficiently between the factors and thus could not be assigned to one single factor. In addition, only items representing the factor well enough, thus loading above 0.5 on one of the factors, were included. This resulted in the deletion of three more items so that in total 15 items were not included in our further analyses.

The final principal component analysis using varimax rotation with the remaining 24 items revealed five factors. However, one factor was not reliable with a Cronbach's alpha value below 0.5; therefore, the final scale includes four factors and 21 items (Table 4). All of the items loaded above 0.5 on one of the factors. The items had a Kaiser-Meyer-Olkin measure of sampling adequacy (KMO) of 0.93, which indicates that the pattern of correlations is very compact, so that the data should produce distinct and reliable factors (49). Item analyses revealed a good—almost excellent—internal consistency of the entire scale with a Cronbach's alpha value of 0.90. The four factors explained in total 58% of the variance. Factor 1 explained 20.7%; factor 2 explained 13.9%; factor 3 explained 11.8%; and factor 4 explained 11.7%. The four factors resulting from the PCA are shown in Table 4. All of the factors could be interpreted in a meaningful way, which is described in the following paragraphs.

**Table 4:** Owner-Bird-Relationship Scale (OBRS) with 21 items (translated into English, the original German formulation is given in parentheses) including means and standard deviations (SD, in parentheses) of scores obtained by a five-point Likert scale.

**Item No**.	**Questionnaire Item**	**Means (SD)**	**Factors**			
			**1**	**2**	**3**	**4**
1	I enjoy playing with my bird (Ich spiele gerne mit meinem Vogel).	3.45 (1.46)	**0.61**	0.13	0.24	0.39
2	I think my bird understands me (Ich bin der Meinung, dass mein Vogel mich versteht).	2.95 (1.34)	**0.66**	0.13	0.10	0.37
3	My bird knows when I'm feeling bad (Mein Vogel weiß, wann es mir schlecht geht).	2.55 (1.43)	**0.66**	0.13	0.06	0.38
4	I consider my bird to be a friend (Ich betrachte meinen Vogel als einen Freund).	3.43 (1.44)	**0.70**	0.28	0.21	0.21
5	My bird is an equal member of my family (Mein Vogel ist ein gleichberechtigter Teil meiner Familie).	3.30 (1.48)	**0.76**	0.18	0.22	0.17
6	Sometimes I wonder what my bird is thinking (Manchmal frage ich mich, was mein Vogel wohl gerade denkt).	3.83 (1.36)	**0.61**	0.13	0.39	−0.01
7	I can talk to my bird about anything (Mit meinem Vogel kann ich über alles reden).	2.47 (1.51)	**0.69**	0.29	0.05	0.09
8	My bird is like a child to me (Mein Vogel ist wie ein Kind für mich).	2.46 (1.50)	**0.72**	0.26	0.04	0.09
9	My bird provides structure for my life (Mein Vogel gibt meinem Leben eine Struktur).	3.26 (1.32)	0.23	**0.71**	0.01	0.07
10	Having a bird gives me something to care for (Einen Vogel zu besitzen gibt mir etwas um das ich mich Kümmern kann).	3.73 (1.25)	0.21	**0.73**	0.09	−0.03
11	My bird makes me feel needed (Durch meinen Vogel fühle ich mich gebraucht).	3.28 (1.35)	0.37	**0.70**	0.04	0.05
12	Spending time with my bird makes me forget my problems for a while (Mich mit meinem Vogel zu beschäftigen, lenkt mich von meinen Problemen ab).	3.60 (1.37)	0.17	**0.68**	0.12	0.13
13	I feel relaxed / more content because of my bird (Durch meinen Vogel bin ich ausgeglichener und zufriedener).	3.92 (1.12)	0.10	**0.69**	0.15	0.16
14	I feel distressed when my bird is ill and I see it suffering (Es belastet mich, wenn mein Vogel krank ist und ich ihn leiden sehe).	4.62 (0.83)	0.10	0.22	**0.60**	0.07
15	When my bird is ill, it is my duty to care for it (Wenn mein Vogel krank ist, ist es meine Pflicht mich um ihn zu kümmern).	4.92 (0.41)	−0.13	0.17	**0.60**	0.10
16	I pay attention to my bird's body language (Ich achte auf die Körpersprache meines Vogels).	4.61 (0.75)	0.24	0.04	**0.68**	0.09
17	My bird has its own unique personality (Mein Vogel hat seine ganz eigene Persönlichkeit).	4.65 (0.81)	0.37	0.05	**0.64**	0.10
18	My bird is a sensitive being with its own needs (Mein Vogel ist ein empfindsames Lebewesen mit seinen eigenen Bedürfnissen).	4.75 (0.63)	0.23	0.04	**0.71**	0.01
19	My bird actively tries to be close to me (Mein Vogel sucht von sich aus meine Nähe).	3.33 (1.41)	0.24	0.13	0.10	**0.79**
20	My bird always keeps a little distance from me (Mein Vogel hält immer ein bisschen Abstand von mir).	3.26 (1.38)	0.14	−0.02	−0.01	**0.80**
21	My bird ignores me (Eigentlich ignoriert mich mein Vogel).	3.96 (1.22)	0.18	0.08	0.07	**0.77**
	Rotated sum of squares loadings		4.33	2.92	2.47	2.46
	% variance		20.7	13.9	11.8	11.7
	Cronbach's alpha subscales		0.89	0.80	0.71	0.76
	Cronbach's alpha scale	0.90				

*Factor loadings above 0.5 are in bold*.

*Results based on questionnaires of 1,444 bird owners. The four factors were labeled “bird as human” (factor 1), “social support” (factor 2), “empathy, attentiveness, and respect” (factor 4), and “relationship of the bird toward the owner” (factor 4). Five-point Likert scale from “strongly disagree” (1) to “strongly agree” (5). Factor loadings after principal component analysis with varimax rotation*.

Eight items showed high loadings on factor 1, all of them reflecting anthropomorphic tendencies. The owner sees his or her bird as an equal interacting partner who understands the human and with whom he or she can talk about everything. The bird is regarded as a human. This factor was labeled “bird as human” and was interpreted as reflecting the dimension of anthropomorphism in the bird owner's relationship to his or her bird. The reliability of the subscale on factor 1 was Cronbach's alpha = 0.89, indicating good reliability.

Five items showed high loadings on factor 2, all of them referring to social support. This is understood as the bird giving structure to the life of the owner and providing an opportunity for the owner to care for someone. This interaction provides the owner with a distraction from her problems; consequently, the owner feels emotionally supported and contented. The reliability of the subscale on factor 2 was Cronbach's alpha = 0.80, indicating good to acceptable reliability.

Another five items showed high loadings on factor 3, all of them referring to the bird as a feeling subject. The owner shows responsibility, cares about the bird in case of illness, and pays attention to the bird's behavior. She or he considers the bird to be a sensitive being with a unique personality. This factor may be labeled “empathy, attentiveness, and respect.” The reliability of the subscale on factor 3 was Cronbach's alpha = 0.71, indicating acceptable reliability.

All three items loading on factor 4 addressed the behavior of the bird toward the owner. They reflect whether the bird seeks to be physically close to the owner or seems uninterested. This factor may be labeled “relationship of the bird toward the owner.” The reliability of the subscale on factor 4 was Cronbach's alpha = 0.76, indicating acceptable reliability.

### Items of the Owner-Bird-Relationship Scale (OBRS)

The mean scores and standard deviations for each item of the final OBRS using the five-point Likert scale [ “strongly disagree” (1) to “strongly agree” (5)] are shown in Table 4. Means > 4.6 and standard deviation < 0.9 for all items loading on factor 3 indicate high empathy, attentiveness, and respect toward the bird (items 14–18). Means > 3.3 and standard deviation between 1.1 and 1.4 for all items loading on factor 2 indicate a somewhat smaller and more varying but nevertheless evident agreement to the social support the bird provides for the bird keeper (items 9–13). Quite similarly, means > 3.3 and standard deviation between 1.2 and 1.4 for all items loading on factor 4 indicate that in the owners' perceptions, most birds show a proximity-seeking behavior toward the owner (items 19–21). In contrast, the items loading on factor 1 show lower means (ranging from 2.6 to 3.8) and higher standard deviations (ranging from 1.3 to 1.5), reflecting the variance of agreement and disagreement to anthropomorphizing birds (items 1-8). In particular, there is considerable disagreement regarding the bird as an interlocutor (item 7) or as a child (item 8).

### Variation of Factors With Owner's Socio-Demography and Bird Group

Descriptive analysis also showed that the estimated individual values of the latent factors for each person (factor scores), that is, the dimensions of the owner-bird relationship, vary with the owner's socio-demography as well as with the bird group. Table 3 shows the average factor scores for seven respondent demographic variables and the bird group (z-standardization transforms the factor scores into values with a mean of 0 and a standard deviation of 1). Z-standardized means < 0 indicate a lower average loading for a respondent group, z-values >0 show a higher than average loading in the group. Z-standardized standard deviations < 1 indicate a lower average variance of the loadings of a respondent group, while z-values >1 show a higher than average variance in the group. Thus, the main advantage of z-standardization is that all factors can be compared with respect to the mean using the average value as a comparison.

Several characteristics of the bird owners were related to a significantly lower tendency to anthropomorphize the bird: male bird owners, bird owners older than 26 years, bird owners with higher levels of education, bird owners with an income higher than 3,000 Euros net per month per household, married bird owners, and bird owners living in rural areas showed less anthropomorphizing behavior (factor 1). The pattern for social support provided by the bird (factor 2) was quite similar. However, there was no significant difference for this factor between the sexes or between bird owners living in rural or urban areas tested with Scheffé tests. Males, bird owners older than 65 years, bird owners with less than tertiary education, and bird owners living in East Germany or living in rural areas showed less empathy, attentiveness, and respect toward their bird (factor 3). The birds—as perceived by the respondents—showed more proximity-seeking behavior toward the bird keeper if s/he was married or lived in West Germany (factor 4).

Viewed from the perspective of the owners' demographic characteristics, gender differences were significantly pronounced with respect to anthropomorphism (factor 1) and empathy, attentiveness, and respect (factor 3); being married reduced the tendency to anthropomorphize (factor 1) or to seek social support from the bird (factor 2). Owners with higher levels of education and income showed a significantly lower tendency to anthropomorphize the bird (factor 1) or to seek social support from it (factor 2).

As mentioned above, the number of birds kept varied among the participants of the study (4% of the participants owned only one bird, 21% had two of them, and 75% kept three or more pet birds). A possible explanatory influence on the owner-bird relationship was not examined here but will be object of future research.

Pet owners more often anthropomorphized (factor 1) their parrots and parakeets, and they also showed more empathy, attentiveness, and respect (factor 3) toward them. However, parrots and parakeets were less often related to social support (factor 2). Finches were the only group of birds in the sample that do not seek physical closeness to the owner (factor 4). The other bird groups constituted a homogeneous subset, and these birds sought to be close to the owner with the exception of the parrots and parakeets, which showed almost no effect.

We also tested possible interactions of the independent variables and found that bird groups should be separately viewed by gender. Women anthropomorphized (factor 1) their parrots and parakeets and their finches much more than men, whereas the gender difference with respect to the other bird groups was smaller. Men showed less empathy, attentiveness, and respect (factor 3) toward finches, ornamental fowl and other bird groups than women, whereas gender differences pertaining to parrots and parakeets were smaller.

### Empirical Owner-Bird Relationships

Referring to our concept of human-animal relationship, the measurement was based on a total of 18 items in the questionnaire and *N* = 1,444 participants. Items measured the five dimensions: series of interactions (D1), personal identity (D2), reciprocity (D3), emotional bond (D4), and empathy (D5). The five dimensions were measured using items on a five-point Likert scale [“strongly disagree” (1) to “strongly agree” (5)]: series of interactions (D1), personal identity (D2), reciprocity (D3), emotional bond (D4), and empathy (D5). These items were part of the 39 items of the survey, so no additional questions were needed. Definitions of the five dimensions were obtained from the literature. Each item was either assigned to one—or if plausible two—dimensions or removed from further consideration. For each group of items assigned to one dimension, inter-item correlation matrices and reliability analyses were calculated. Items with insufficient reliability on the subscales were removed. Item analyses revealed a satisfactory internal consistency of the five scales with a Cronbach's alpha value of 0.78 (D1 scale), 0.66 (D2 scale), 0.69 (D3 scale), 0.88 (D4 scale), and 0.79 (D5 scale). In order to be classified as either a personal or a close personal relationship, the measures had to exceed a certain threshold: This threshold was set to 4 on the 5-point-Likert scale for the close personal relationship, meaning that indecisive answers and answers of disagreement were excluded (D2 ≥ 4, D3 ≥ 4, D4 ≥ 4, and D5 ≥ 4). For personal relationships we reduced this threshold on the reciprocity dimension from 4 to 2.5, meaning that it was sufficient for a bird keeper and her bird to be included if the answers on the reciprocity items were either indecisive or moderate disagreement (D2 ≥ 4 and D3 ≥ 2.5).

It was found that 26.0% of the respondents showed an impersonal, 49.8% a personal, and 22.9% a close personal relationship with their bird. While the overwhelming majority of these bird keepers regarded their bird as having a unique personality and showed a high level of empathy toward it, only 29.2% had developed a strong emotional bond to their bird. Half of the birds showed—as perceived by their keepers—a proximity-seeking behavior toward the latter, indicating some kind of reciprocity (measured by items 19 and 21). The emotional bond was thus the important empirical difference of those bird keepers who showed a close human-bird relationship.

In addition, we tested the robustness of the results conducting sensitivity analyses for D3 ≥ 3 (instead of 2.5) and a two- vs. six-item version of the D5 scale. Results were robust: In the D3 ≥ 3 scenario, the proportion of personal relationships dropped moderately from 49.79% to 44.18% and the proportion of impersonal relationships increased moderately from 25.97% to 31.23%. The proportion of close personal relationships did not change. In the two-item version of the D5 scale the proportion of personal relationships showed a minor increase from 49.79% to 51.94%, and the proportion of impersonal relationships a minor increase from 25.97% to 26.25%. The proportion of close personal relationships dropped from 22.92% to 20.78%.

## Discussion

### Similarities in the Human-Bird Relationships Beyond Different Bird Species

The aim of this study was to develop a multidimensional scale to measure the relationship between an owner and his or her bird. Apparently, the owner-bird relationship is as multifaceted as the relationship between an owner and a dog or a cat. Similarly to Zasloff's statement that “a dog is not a cat is not a bird” (19), it had to take into account that a gray parrot is not a chicken is not a canary. It is difficult to account for about 8,800 bird species with more than 28,000 subspecies (8) within a single scale as there is a wide variation not only in terms of anatomy but also in terms of physiology. Even if most birds fly, this does not make them all equal. Some species, such as African gray parrots or common ravens (50, 51), are highly intelligent and need much more interaction, work, and challenges than other species to prevent behavioral disorders. Nevertheless, our interest was in whether there are—despite all the differences among bird species—any similarities in the human-bird relationship as perceived by their keepers which cross the boundaries between species. Similarities in the relationships between the owners of different bird groups were found indeed (Table 3). Yet no specific factor structure for different bird group owners was found other than those explained in the results' section. It has to be noted that for many species the sample did not include enough cases for a separate statistical analysis.

### Anthropomorphism

The most important dimension describing the relationship of the bird owner to her or his pet was the degree to which the owner anthropomorphized the bird (factor “bird as human”). Anthropomorphism in this case means attributing human social motivations to animals (52). Anthropomorphizing bird owners described the bird as a partner for interaction who understands the owner and is considered a friend or family member. The relationship was characterized by features such as interaction, interdependence, reciprocity, emotional closeness, personal identity, self-disclosure, intimacy, and sensibility for others. Similar factors with anthropomorphic items were described in former studies for dogs (53) and general pet keeping (34, 37). Participants in the study by Anderson (9) mentioned, for example, “birds as family/children/Fids” and “companionship” in the top 10 responses describing the rewards of avian companionship. Motives such as “anthropomorphism” and “companionship” were described for tortoises as well (54). This is of outstanding relevance because the attribution of human feelings to animals can influence animal welfare (18, 52, 55, 56).

### Social Support

The second important dimension was classified as social support. The bird not only provides a structure for the owner's daily life (activities such as feeding, cleaning, watching, or playing all follow a defined schedule). In addition, the items indicated that the owner feels needed and is distracted from his or her problems by the bird. Those bird owners who experienced social support from the bird developed relationships that were characterized by the authority of the owner and the co-dependence of or on the bird. Social support by the animal is a factor that several previous studies have already identified (18, 20, 29, 30, 34, 53). Our study supports the findings of (18) of social support as a main model to explain the affiliation of humans to birds. About 1 out of 4 respondents (27%) of this investigation gave answers that indicated that their bird provides social support.

### Empathy, Attentiveness, and Respect

The third dimension describing the relationship of the bird owner to his or her pet is the degree to which the owner showed empathy, attentiveness, and respect toward the bird. Empathetic, attentive, and respectful bird owners perceived the bird as a sentient individual and watched the body language of the bird. Their relationship to the bird was characterized not only by empathy but also by the attribution of a personal identity, by emotional closeness, and the perception of uniqueness. This is a major difference between this study and previous studies on dogs or cats, which simply presumed the attribution of a personal identity and feelings to the companion animal. It is important to recognize that birds of many species are typically not considered as individuals in general discussions (57). The finding that empathy, attentiveness, and respect toward the bird was not unique to parrots but could be shown for all bird groups. However, there is a significant difference between bird groups, parrots, and parakeets scoring higher than finches and ornamental fowl. Respect and attentiveness have also been found in studies of other pet animals, such as dogs and horses (22, 58).

### The Bird's Relationship to the Bird Keeper

In this study, the relationship between a bird owner and the bird was investigated not only from the perspective of the human. It has to be noted that there were two populations in this study: the bird owners and the pet birds. Of course, only the bird keepers answered the questionnaire; thus, they constitute the only sources of knowledge. But the questions were designed to address both populations. As in the case of small children who cannot themselves answer the questions of social researchers, birds and their behavior were empirically explored by questions addressed to the bird keeper (Table 3, items 19–21). Factor 4 can be interpreted as a proxy for the relationship of the bird vis-à-vis her keeper. The factor analysis revealed the relationship of the bird toward the owner as a fourth dimension of the bird-owner relationship. This dimension can be interpreted as including an aspect of gratuitousness as well as autonomy and therefore refers to the characteristic of reciprocity in the relationship. In some human-bird relationships specific norms and habits are formed which are also indicators of reciprocity. The reported behavior of the bird may also reflect an emotional bond toward the bird owner on the part of the bird, but exploring this bond goes beyond the scope of our study.

Considering the statistical method, it is interesting that the perspective of the bird had such an importance: the factor analysis revealed the order of relevance of each of the factors. The proximity-seeking behavior of the bird toward the owner turned out to be the fourth strongest factor. Consequently, it should be given more attention in future studies into human-bird relationships. To gain more insight into the bird's perspective, further studies should include behavioral observation of the birds at home.

### Limitations

There are several limitations associated with this study. We will focus on the representativeness and generalizability of the results.

First, a convenience sample was used to generate data—a specific type of non-probability sample that relies on data collection from population members who are conveniently available to participate in a study. Thus, the sources which had to be relied on, such as in Facebook pools—contained a heterogeneous population in order to recognize a wide range of possible owner-bird relationships. Since data on the pet bird owner population in Germany are not available, the representativeness of the sample of this study cannot be evaluated. Nevertheless, a major German representative study was used for a conservative estimate of the selectivity of our sample (“German General Social Survey (GGSS)—Allgemeine Bevölkerungsumfrage der Sozialwissenschaften (ALLBUS)”) (59). The comparison was limited by the use of different categories in the reference data. In comparison to the German population, our participants were more often female, younger, and single. In particular, males, people over 60 years, and households with children were somewhat underrepresented. The poor participation of elderly people can be attributed to the use of an online questionnaire. Furthermore, the participants showed a higher level of education compared to the general population and were more often employed. However, it is quite possible that the bird-owning population differs from the general population in Germany. For the purpose of this study, a heterogeneous sample was needed to allow for identifying different human-bird relationships. We have endeavored in several ways to ensure that our sample is heterogeneous. For example, the participants differed in their socio-demographic characteristics, and they owned bird species belonging to all groups of pet birds. It has to be expected that there has been at least one systematic selective effect—a positive self-selection into the sample. Participants who do not regard their pet birds as important enough to report about them were probably missed. It is likely that the recruiting methods used here were unable to reach owners who are not interested in activities with or about their birds. Moreover, owners who do not feel close to their bird may not be motivated to fill in a 20-min questionnaire about it. Whether the number of birds the participants of this investigations owned had an influence on the results of this study, remains an open question for further research. Therefore, the distribution of empirical human-animal relationships shown here applies particularly to the sample of this survey and cannot be generalized to all German bird owners. In particular, we would expect a higher number of owners with an impersonal owner-bird relationship in the empirical population of bird owners.

Second, the scale was developed for German-speaking countries, especially Germany. In order to be used in other countries, it would need to be adapted and tested in future studies considering country specific as well as cultural differences. This requires linguistic as well as cultural equivalence because the human-animal relationship must always be considered within the context of the dominant religion, culture, and society (60). Therefore, the scale may yield different results in different cultures or may even be inappropriate to describe some features of the local owner-bird relationships and thus require adaptation. However, the population in countries such as the United States of America or Canada share with the German population major societal developments of late modernity (such as individualization) and a common cultural and religious heritage, which would seem to support the usability of the OBRS scale in these countries. However, differences in, for example, specific government regulations for some bird species may influence the knowledge of the owner about bird keeping and therefore the manifestation of the different factors. In Germany, there are specifications for the capture of raptors, similar to the different restrictions on falconry in the United States of America. In this study, a difference between such birds and other bird species was not found, yet we are limited by the low number of participants reporting on their raptors (*N* = 36).

## Conclusion

Up till now, no psychometric scale to measure the human-avian-relationship has ever been devised. The results of this study indicate that the owner-bird-relationship scale (OBRS) shows a promising attempt in measuring the relationship between the owner and his or her pet birds (Cronbach's alpha = 0.90). This scale can be used in standardized empirical studies to measure the relationship between a bird and its owner. Further applications of the scale include bird welfare studies and studies about the adherence and compliance of bird owners in veterinary therapy. To test and further develop the scale as well as to add to our knowledge on the owner-bird relationship, for example across bird species and cross-culturally, the items need to be translated and adjusted for different languages and cultures.

The findings of this study provide an important insight into the characterization of relationships between owners and their pet birds in non-commercial bird-keeping. The basic requirements for a relationship (interdependence, interaction, and personal identity) were met, and the existence of a relationship between the human and the pet bird could be confirmed. This relationship varies and is multifaceted. The owner-bird relationship was found to involve four dimensions: the tendency of the owner to anthropomorphize the bird; the social support the owner receives from the bird; the empathy, attentiveness, and respect of the owner toward the bird; and the relationship of the bird toward the owner. The relationship with birds is revealed to be just as complex as the relationship with “typical” pets, such as dogs or cats. So while “a dog is not a cat is not a bird” (19), what they all have in common is the absence of a uniform relationship. Every relationship between a human and a companion animal appears to be as unique as the two individuals involved. This confirms the argument by Bergler, Hoff (22) that the human-animal relationship always depends on the species as well as the breed and the individual type. However, there may be a limited number of patterns behind this variability, which is a topic for further studies.

Finally, as outlined above, our sociological concept of human-animal relationships requires behavioral observations of the pet animals in order to understand the animal's relationship to its keeper. We need a collaboration between behavioral biologists and sociologists to learn more about the relationship from the point of view of the pet animal.

## Data Availability Statement

The raw data supporting the conclusions of this article will be made available by the authors, without undue reservation.

## Ethics Statement

Ethical review and approval and written informed consent were not required for the animal study because the authors performed an online survey. The bird owners answered questions about their birds.

## Author Contributions

A-KB, NS, RK, MR, and AP designed the study. A-KB executed the study. A-KB, KD, SP, and NS analyzed the data. A-KB, KD, MR, SP, RK, and NS interpreted the data. A-KB, KD, MR, AP, RK, and NS were involved in the writing and revising of the paper. All authors contributed to the article and approved the submitted version.

## Conflict of Interest

The authors declare that the research was conducted in the absence of any commercial or financial relationships that could be construed as a potential conflict of interest.
